# Implementation of Robson’s ten-group classification system for cesarean section rates at a tertiary university hospital in Egypt: a prospective study

**DOI:** 10.1186/s12884-026-09693-y

**Published:** 2026-07-23

**Authors:** Ahmed Gamal Ahmed Alamrawy, Tarek Abdel-Zaher Karkour, Tamer Mamdouh Abdel-Dayem, Hesham Adel El Fazary

**Affiliations:** Department of Obstetrics and Gynecology, Faculty of Medicine, Alexandria University, Alexandria, Egypt

**Keywords:** Cesarean section, Robson classification, Egypt, TOLAC, VBAC, Clinical audit, Quality improvement, Tertiary hospital, Preterm birth

## Abstract

**Background:**

Egypt has one of the highest reported cesarean section (CS) rates globally. Aggregate CS rates alone, however, do not identify which obstetric populations contribute most to CS use or whether interventions are clinically appropriate. The Robson Ten-Group Classification System (TGCS) provides a standardized method for institutional audit and benchmarking.

**Methods:**

We conducted a prospective observational study at El-Shatby Maternity University Hospital, Alexandria, Egypt, from September 1, 2021, to March 1, 2022. All women admitted for delivery at ≥ 28 weeks’ gestation and/or fetal weight > 500 g were included. Women were categorized into Robson groups using the six core obstetric variables. Proportions and 95% confidence intervals (CIs) were calculated for the main estimates.

**Results:**

Among 5682 deliveries, 3405 were by CS (59.9%, 95% CI 58.6–61.2). Group 5 was the largest group (1742/5682, 30.7%) and contributed 47.6% of all CSs, with a group-specific CS rate of 93.1% (95% CI 91.8–94.2). Group 10 comprised 16.7% of the obstetric population and contributed 20.6% of all CSs; its group-specific CS rate was 73.9% (95% CI 71.1–76.6). Groups 2 and 4 had CS rates of 47.1% and 50.4%, respectively. Group 1 and Group 3 had lower group-specific CS rates of 14.5% and 11.9%, respectively. Vaginal birth after cesarean (VBAC) occurred in 126 cases (2.2% of all deliveries).

**Conclusions:**

This prospective audit established a standardized Robson baseline for a high-volume Egyptian tertiary maternity unit. The high overall CS rate reflected a large Group 5, high CS rates in modifiable Groups 2 and 4, and a substantial contribution from Group 10. The findings identify priorities for future audit, including prevention of unnecessary primary CS, assessment of trial of labor after cesarean (TOLAC) eligibility and counseling, and review of preterm CS decision-making. They should not be interpreted as proving that all observed CSs were unnecessary.

**Supplementary Information:**

The online version contains supplementary material available at 10.1186/s12884-026-09693-y.

## Background

Cesarean section (CS) is a life-saving intervention when clinically indicated, but rising use beyond medical need is a major public-health and health-system concern [1–3]. Egypt has reported very high national CS rates, including 72% in the Egypt Family Health Survey 2021 [4]. Repeated or non-medically indicated CS can increase future obstetric risk, particularly placenta accreta spectrum disorders, and can impose avoidable economic burden on health systems and families [5–8]. Such rates require careful interpretation, because an institutional CS rate may be influenced by referral patterns, case-mix, patient preference, provider thresholds, medico-legal concerns, and availability of alternatives to CS.

A high CS rate alone does not demonstrate poor-quality care or inappropriate intervention. To improve care, hospitals require a standardized method that identifies which obstetric populations contribute most to CS use and that allows comparisons across time and between facilities. The World Health Organization (WHO) therefore recommends the Robson Ten-Group Classification System (TGCS), which classifies all women admitted for delivery into 10 mutually exclusive and totally inclusive groups based on parity, previous CS, onset of labor, number of fetuses, gestational age, and fetal lie/presentation [9, 10].

The TGCS is particularly useful as an audit and benchmarking tool because it separates CS use into clinically interpretable groups. It can help distinguish between the consequences of previous primary CSs, such as Group 5, and potentially modifiable current-care decisions in Groups 1–4, including induction and pre-labor CS. However, the TGCS does not by itself determine whether an individual CS was appropriate; such an assessment requires indication review, maternal and neonatal outcomes, and local clinical context.

The global rise in CS rates and Egyptian multi-governorate data reinforce the need for standardized institutional monitoring [11, 12]. Implementation of the TGCS in Egyptian tertiary centers remains limited. El-Shatby Maternity University Hospital is a tertiary referral unit serving Alexandria and the surrounding governorates. Establishing a local Robson baseline is a necessary first step for longitudinal monitoring and future quality-improvement work.

The aim of this study was to prospectively implement the Robson TGCS at El-Shatby Maternity University Hospital, describe group-specific CS rates and contributions, and identify priority groups for future audit and quality-improvement initiatives. The study was designed as a descriptive institutional audit and was not intended to establish causality or judge the appropriateness of individual CS decisions.

## Methods

### Study design and setting

This was a prospective observational institutional audit conducted at El-Shatby Maternity University Hospital, Faculty of Medicine, Alexandria University, Egypt, from September 1, 2021, to March 1, 2022. The hospital is a high-volume tertiary referral maternity unit that manages low-risk and high-risk obstetric cases from Alexandria and nearby governorates. During the six-month audit period, 5682 deliveries were recorded, equivalent to an annualized caseload of approximately 11,364 deliveries. Comparable five-year Robson-classified CS trend data were not available before this implementation.

### Participants

All women admitted for delivery during the study period were eligible. Inclusion criteria were admission for delivery (spontaneous labor, induced labor, or pre-labor CS) with gestational age ≥ 28 weeks and/or fetal weight > 500 g. During prospective data collection, pregnancies meeting the birth-weight criterion despite a gestational age of < 28 weeks would have been eligible for inclusion according to the study protocol. However, no such cases were encountered during the study period. Consequently, all included participants were ≥ 28 weeks’ gestation, and birth weight was not retained as a study variable in the final dataset because it was not required to determine eligibility for any enrolled case. There was no maternal age restriction. Cases below the local threshold for fetal viability were not included.

### Data collection and quality assurance

Data were collected prospectively using a standardized data collection sheet (Supplementary File 1). The core Robson variables recorded for each woman were parity, previous CS, onset of labor, number of fetuses, gestational age, and fetal lie/presentation. Mode of delivery and recorded CS indications or associated clinical factors were also collected.

A team of obstetrics residents collected data from admission records, labor-ward records, operative notes, discharge records, and the hospital registry. Before data collection, residents attended a two-day orientation on Robson definitions, group allocation logic, and completion of the data form. Ambiguous cases were discussed with the supervising authors. The lead author reviewed data sheets regularly for missing or inconsistent core variables. When a core variable was incomplete or inconsistent, the patient record was re-examined and, when needed, clarification was obtained from the treating resident or operating surgeon. The final dataset was cross-checked against the hospital delivery registry. No woman was unclassifiable for the six core Robson variables.

During electronic database construction, the Robson group assigned in the clinical records was verified by the principal investigator using the six core Robson variables. Whenever discrepancies were identified, the original clinical records were rechecked and the classification was corrected before final database locking.

### Robson classification and definitions

**Fig. 1:**
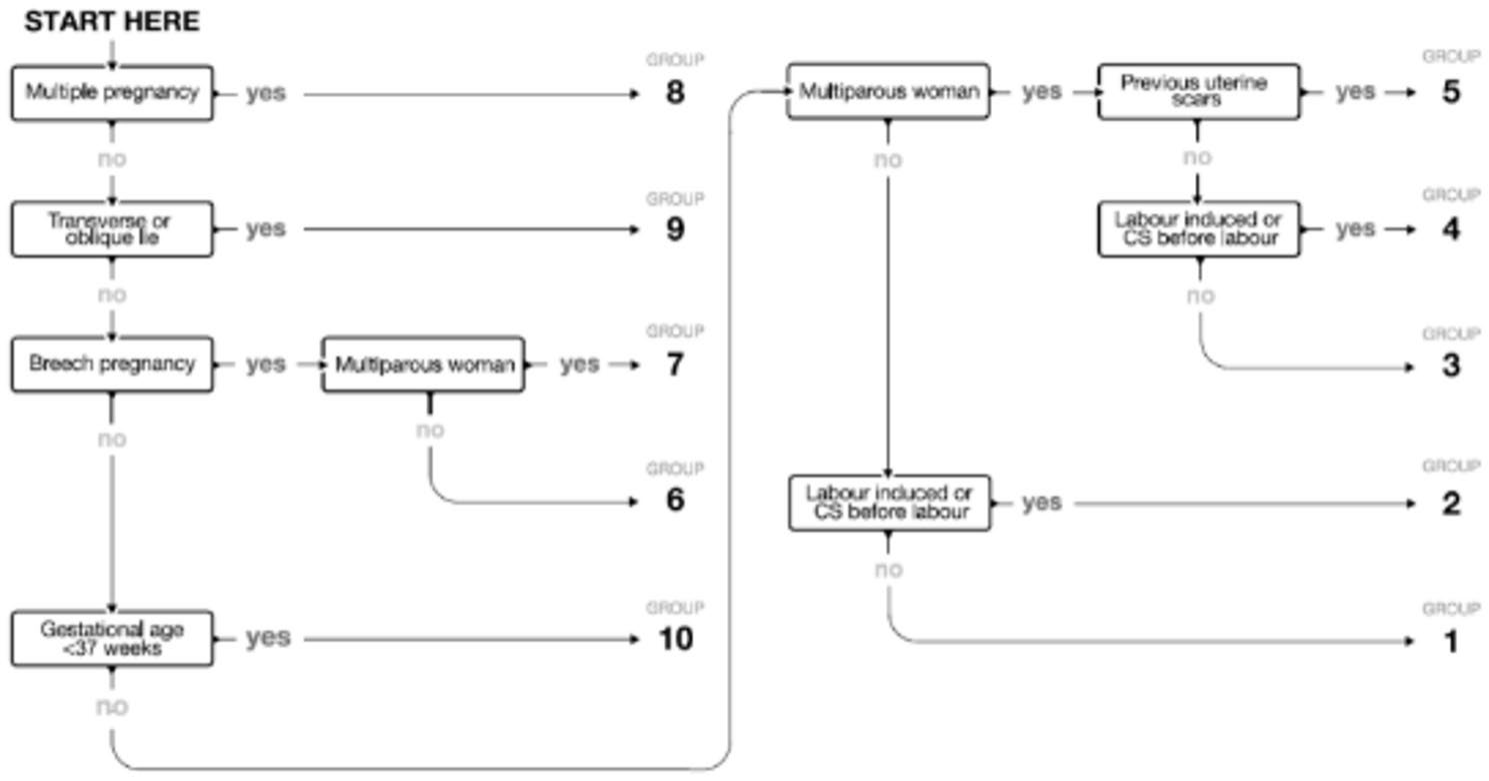
Flow chart for assigning women to Robson groups

Women were assigned to one of the 10 Robson groups according to Robson’s original classification and WHO implementation definitions [10, 13] (Fig. 1). Groups 2 and 4 were subdivided into induced labor (2a/4a) and pre-labor CS (2b/4b). Group 10 included all singleton cephalic pregnancies < 37 weeks’ gestation, including women with previous CS. Group size, group-specific CS rate, absolute contribution, and relative contribution were calculated according to the standard Robson report table.

- Group size (%) = number of women in the group / total number of women delivered × 100.
- Group-specific CS rate (%) = number of CSs in the group / total number of women in the group × 100.
- Absolute contribution (%) = number of CSs in the group / total number of women delivered × 100.
- Relative contribution (%) = number of CSs in the group / total number of CSs × 100.

The WHO Robson classification flow chart was used to assign each woman to one mutually exclusive group according to the number of fetuses, fetal lie/presentation, gestational age, parity, previous CS, and onset of labor. The original data collection sheet is provided as Supplementary File 1.

### Recorded indications/associated clinical factors and data validity

Recorded indications and associated clinical factors for cesarean section were extracted exactly as documented in the routine clinical records by the treating team. Specific clinical findings, such as “tender scar,” were extracted exactly as documented by the treating obstetrician in the medical record and were not independently redefined by the investigators. More than one recorded indication or associated clinical factor could be assigned to the same CS. Consequently, Table 3 should be interpreted as a description of recorded indications/associated clinical factors rather than mutually exclusive dominant indications. Percentages within each Robson-group column use the number of CSs in that group as the denominator and may sum to more than 100%. Independent blinded validation of recorded indications, cardiotocography (CTG) tracings, or operative notes was not performed.

### Statistical analysis

Data were analyzed using IBM SPSS Statistics version 20.0 (IBM Corp., Armonk, NY, USA) and rechecked against the original dataset during revision. Qualitative variables are presented as numbers and percentages. Quantitative variables are presented as range, mean, standard deviation, and median. 95% confidence intervals (CIs) for proportions were calculated using the Wilson score method. Analyses were strictly descriptive; no causal modeling or comparative inferential statistics were performed.

### Ethics approval and consent to participate

The study was approved by the Ethics Committee of the Faculty of Medicine, Alexandria University (IRB number: 00012098; FWA number: 00018699; serial number: 0106875) on August 19, 2021. Because the study was an observational audit of routinely collected clinical data, the requirement for informed consent was waived by the Ethics Committee. All methods were carried out in accordance with relevant guidelines and regulations, including the Declaration of Helsinki.

## Results

During the study period, 5682 women were admitted for delivery and included in the analysis. CS was performed in 3405 cases (59.9%, 95% CI 58.6–61.2), and 2277 women (40.1%) delivered vaginally. Vaginal birth after cesarean (VBAC) occurred in 126 cases, representing 2.2% of all deliveries and 5.5% of vaginal deliveries.

### Demographic and obstetric characteristics

Table 1 shows the demographic and obstetric characteristics of the study population. The onset-of-labor values were rechecked against the raw dataset during revision: 4476 women had spontaneous labor, 510 had induced labor, and 696 had pre-labor CS. The previous-CS subsection in Table 1 refers only to parous women (*n* = 4178).

**Table 1:** Demographic and obstetric characteristics of the study population (*n* = 5682)

Variable	Total *n* (%)	VD *n* (%)	CS *n* (%)
Maternal age (years)			
<=20	828 (14.6)	509 (61.5)	319 (38.5)
> 20–25	960 (16.9)	500 (52.1)	460 (47.9)
> 25–30	1278 (22.5)	328 (25.7)	950 (74.3)
> 30–35	1368 (24.1)	497 (36.3)	871 (63.7)
> 35–40	995 (17.5)	378 (38.0)	617 (62.0)
> 40	253 (4.5)	65 (25.7)	188 (74.3)
Mean ± SD (range)	29.29 ± 6.78 (14–42)	27.75 ± 7.23 (14–42)	30.31 ± 6.25 (17–42)
Onset of labor			
Spontaneous labor	4476 (78.8)	1894 (42.3)	2582 (57.7)
Induced labor	510 (9.0)	383 (75.1)	127 (24.9)
Pre-labor CS	696 (12.2)	0 (0.0)	696 (100.0)
Parity			
Nulliparous	1504 (26.5)	848 (56.4)	656 (43.6)
Parous	4178 (73.5)	1429 (34.2)	2749 (65.8)
Previous CS status among parous women only (*n* = 4178)			
No previous CS	1947 (46.6)	1303 (66.9)	644 (33.1)
One previous CS	1176 (28.1)	126 (10.7)	1050 (89.3)
>=2 previous CS	1055 (25.3)	0 (0.0)	1055 (100.0)
Gestational age			
< 37 weeks	1043 (18.4)	308 (29.5)	735 (70.5)
>=37 weeks	4639 (81.6)	1969 (42.4)	2670 (57.6)
Number of fetuses			
Singleton	5288 (93.1)	2150 (40.7)	3138 (59.3)
Twin pregnancy	387 (6.8)	127 (32.8)	260 (67.2)
Triplet pregnancy	7 (0.1)	0 (0.0)	7 (100.0)
Fetal presentation			
Cephalic	5015 (88.3)	2117 (42.2)	2898 (57.8)
Breech	599 (10.5)	160 (26.7)	439 (73.3)
Transverse/oblique	68 (1.2)	0 (0.0)	68 (100.0)

Percentages for the VD and CS columns are row percentages within each variable category. For the previous-CS subsection only, Total n (%) is calculated among parous women (*n* = 4178)

*CS* Cesarean section, *VD* Vaginal delivery, *SD* Standard deviation

### Robson classification analysis

**Fig. 2:**
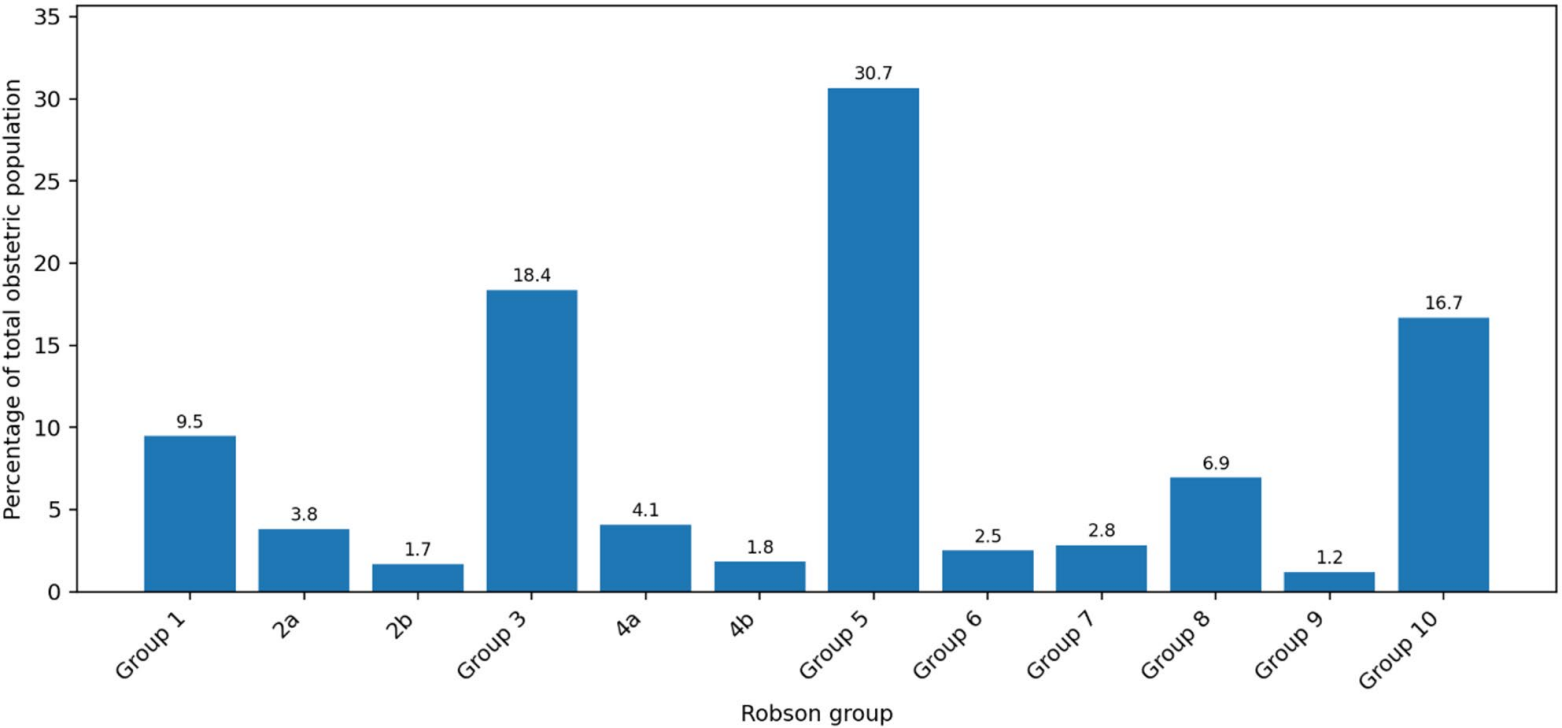
Distribution of obstetric population by Robson group size

**Fig. 3:**
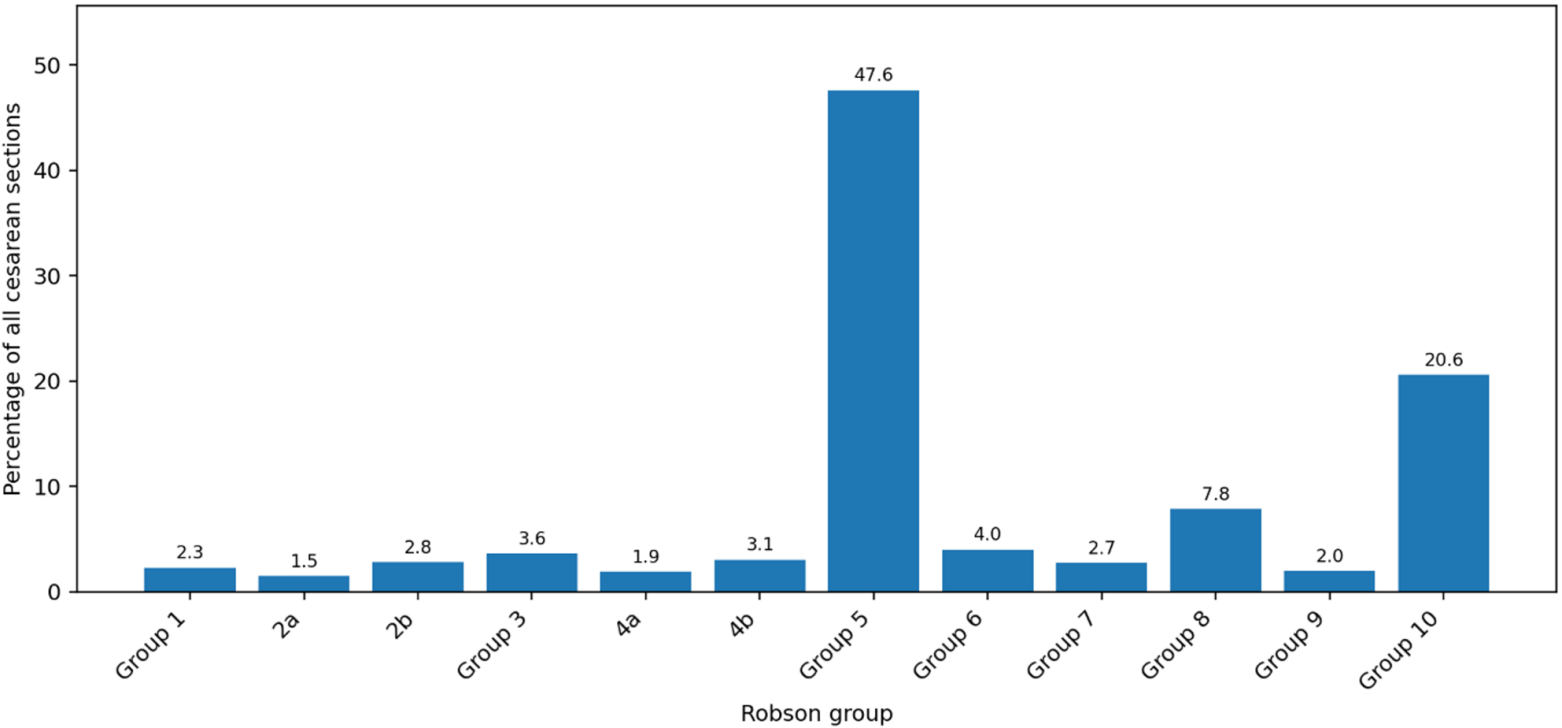
Relative contribution of each Robson group to the total number of CSs

Table 2 presents the Robson classification report table. Group 5 was the largest group (30.7% of the obstetric population; Fig. 2) and contributed 47.6% of all CSs (Fig. 3). Group 10 represented 16.7% of the obstetric population (Fig. 2) and contributed 20.6% of all CSs (Fig. 3). Groups 2 and 4 had group-specific CS rates of 47.1% and 50.4%, respectively; within these groups, pre-labor CS subgroups 2b and 4b had CS rates of 100% by definition. Group 1 and Group 3 had group-specific CS rates of 14.5% and 11.9%, respectively.

**Table 2:** Robson group size, group-specific CS rate, absolute contribution, and relative contribution

Robson group	CS in group	Women in a group	Group size (%)	Group CS rate % (95% CI)	Absolute contribution (%)	Relative contribution (%)
Group 1	78	538	9.5	14.5 (11.8–17.7)	1.4	2.3
Group 2	147	312	5.5	47.1 (41.6–52.7)	2.6	4.3
2a: induced labor	51	216	3.8	23.6 (18.4–29.7)	0.9	1.5
2b: pre-labor CS	96	96	1.7	100.0 (96.2–100.0)	1.7	2.8
Group 3	124	1043	18.4	11.9 (10.1–14.0)	2.2	3.6
Group 4	169	335	5.9	50.4 (45.1–55.8)	3.0	5.0
4a: induced labor	65	231	4.1	28.1 (22.7–34.3)	1.1	1.9
4b: pre-labor CS	104	104	1.8	100.0 (96.4–100.0)	1.8	3.1
Group 5	1621	1742	30.7	93.1 (91.8–94.2)	28.5	47.6
Group 6	137	142	2.5	96.5 (92.0-98.5)	2.4	4.0
Group 7	93	160	2.8	58.1 (50.4–65.5)	1.6	2.7
Group 8	267	394	6.9	67.8 (63.0-72.2)	4.7	7.8
Group 9	68	68	1.2	100.0 (94.7–100.0)	1.2	2.0
Group 10	701	948	16.7	73.9 (71.1–76.6)	12.3	20.6
Total	3405	5682	100.0	59.9 (58.6–61.2)	59.9	100.0

95% CIs were calculated using the Wilson score method. Group 2 = nulliparous, singleton cephalic, ≥ 37 weeks, induced labor or pre-labor CS; Group 4 = parous without previous CS, singleton cephalic, ≥ 37 weeks, induced labor or pre-labor CS

Within Group 5, 890 women had one previous CS and 852 had two or more previous CSs. Among women with one previous CS in Group 5, 121 delivered vaginally. Eligibility for trial of labor after cesarean (TOLAC) was not prospectively captured. In Group 10, 285 of 701 CSs (40.7%) occurred among women with a previous CS, while 416 (59.3%) occurred among women without a previous CS or among nulliparous women; therefore, Group 10 should be interpreted as a heterogeneous preterm group.

### Recorded indications and associated clinical factors for CS

Table 3 summarizes recorded indications and associated clinical factors for CS within each Robson group. Because multiple factors could be recorded for the same CS, the percentages in Table 3 are not mutually exclusive. Tender scar was recorded in 1275 of 1621 Group 5 CSs (78.7%). In Group 10, the most frequent recorded factors were preeclampsia (231/701, 33.0%), pre-labor CS (216/701, 30.8%), fetal distress (172/701, 24.5%), and oligohydramnios (133/701, 19.0%). A total of 180 CSs (5.3%) had no recorded indication.

**Table 3:** Recorded indications/associated clinical factors for CS by the Robson group (*n* = 3405 CSs)

Recorded indication/factor	Total *n* (%)	G1	G2	G3	G4	G5	G6	G7	G8	G9	G10
Elective/pre-labor CS	696 (20.4)	-	96 (65.3)	-	104 (61.5)	130 (8.0)	116 (84.7)	22 (23.7)	10 (3.7)	2 (2.9)	216 (30.8)
Failed induction	127 (3.7)	-	56 (38.0)	-	71 (42.0)	-	-	-	-	-	-
Missing recorded indication	180 (5.3)	4 (5.1)	4 (2.7)	2 (1.6)	2 (1.2)	78 (4.8)	-	5 (5.4)	-	-	85 (12.1)
Tender scar	1283 (37.7)	-	-	-	-	1275 (78.7)	-	3 (3.2)	5 (1.9)	-	-
Oligohydramnios	174 (5.1)	34 (43.6)	-	5 (4.0)	2 (1.2)	-	-	-	-	-	133 (19.0)
Preeclampsia (PET)	325 (9.5)	-	-	-	-	46 (2.8)	-	48 (51.6)	-	-	231 (33.0)
Obstructed labor	70 (2.1)	6 (7.7)	-	61 (49.2)	3 (1.8)	-	-	-	-	-	-
Placenta previa	154 (4.5)	3 (3.8)	-	-	57 (33.7)	48 (3.0)	-	-	-	-	46 (6.6)
Antepartum hemorrhage (APH)	23 (0.7)	12 (15.4)	5 (3.4)	-	-	-	-	-	-	-	6 (0.9)
Macrosomia	9 (0.3)	3 (3.8)	2 (1.4)	1 (0.8)	1 (0.6)	-	-	-	-	-	2 (0.3)
Previous myomectomy	51 (1.5)	-	-	50 (40.3)	-	-	-	-	-	-	1 (0.1)
Cephalopelvic disproportion (CPD)	83 (2.4)	9 (11.5)	40 (27.2)	-	-	-	-	-	-	-	34 (4.9)
Fetal distress	179 (5.3)	-	-	5 (4.0)	2 (1.2)	-	-	-	-	-	172 (24.5)
Abnormal presentation	314 (9.2)	-	-	-	-	-	81 (59.1)	25 (26.9)	140 (52.4)	68 (100.0)	-
Primary infertility	113 (3.3)	5 (6.4)	49 (33.3)	-	-	-	5 (3.6)	-	-	-	54 (7.7)
Multiple pregnancy	261 (7.7)	-	-	-	-	-	-	-	261 (97.8)	-	-
Fetal congenital anomaly	91 (2.7)	-	-	-	-	91 (5.6)	-	-	-	-	-

Cells show n (%) of CSs in that Robson group with the recorded factor. The denominator for each group column is the number of CSs in that group. Multiple recorded indications/factors could be assigned to the same CS; therefore, column percentages may sum to more than 100%

## Discussion

This prospective institutional audit implemented the Robson TGCS in 5682 deliveries at a high-volume tertiary maternity hospital in Egypt. The main findings were: (1) the overall CS rate was 59.9%; (2) Group 5 was the largest group and contributed nearly half of all CSs; (3) Group 10 was the second largest contributor to all CSs and was heterogeneous, with both previous-CS and non-previous-CS pregnancies represented; (4) Groups 2 and 4 had high group-specific CS rates, highlighting current-care populations that may be modifiable; and (5) Group 1 and Group 3 had lower CS rates than the overall hospital rate.

### Interpretation of the overall CS rate

The overall CS rate is high compared with international population-level benchmarks, but this should be interpreted cautiously because the study was conducted in a tertiary referral hospital. The TGCS helps explain the structure behind the aggregate rate: the hospital’s CS burden reflects a large Group 5, high CS rates in the induced/pre-labor CS groups, and a substantial contribution from preterm cephalic singleton pregnancies. These data identify where additional audits should be targeted; they do not prove that individual CS decisions were inappropriate.

### Group 5 and TOLAC/VBAC

Group 5 accounted for 30.7% of the obstetric population and 47.6% of all CSs. This finding is consistent with an Egyptian Robson analysis showing that previous CS is a major contributor to the overall CS burden [14]. However, Group 5 reflects the downstream consequence of earlier primary CSs; therefore, a strategy focused only on VBAC would be incomplete. The present audit could not determine the proportion of women eligible for TOLAC because scar type, indication for previous CS, interpregnancy interval, estimated fetal weight, contraindications, maternal preference, and counseling documentation were not prospectively captured. Future audits should specifically record TOLAC eligibility, counseling, acceptance, intrapartum monitoring, and outcomes.

### Primary CS and modifiable Groups 1–4

Prevention of unnecessary primary CS is essential for long-term reduction in repeat CS. In this study, Group 1 and Group 3 had relatively lower CS rates (14.5% and 11.9%, respectively), whereas Groups 2 and 4 had CS rates of 47.1% and 50.4%. The 100% CS rate in subgroups 2b and 4b is expected by definition, but their presence emphasizes the need to audit indications for pre-labor CS in women without previous CS. Similarly, induction practices should be reviewed, including indications for induction, cervical status, induction methods, duration of induction, and criteria for failed induction.

### Group 10 and preterm CS

Group 10 contributed 20.6% of all CSs, with a group-specific CS rate of 73.9%. This group should not be interpreted as a single clinical entity. Previous CS accounted for 40.7% of Group 10 CSs, but most Group 10 CSs occurred in women without a previous CS or in nulliparous women. The most frequently recorded factors in Group 10 were preeclampsia, pre-labor CS, fetal distress, and oligohydramnios. Because preterm CS may be medically indicated in severe maternal or fetal disease, and because CS does not universally improve preterm outcomes, these findings support further subgroup audit rather than a conclusion that preterm CS was excessive [15, 16]. Future studies should further characterize Group 10 by gestational-age subgroups, previous cesarean status, and specific obstetric indications to better identify potentially modifiable contributors to the high cesarean section rate in this heterogeneous population.

### Recorded indications/associated clinical factors and appropriateness of CS

Recorded indication and associated clinical factor data provide clinical context, but do not establish appropriateness. Recorded indications such as tender scar, fetal distress, obstructed labor, and oligohydramnios are subject to variation in clinical thresholds and documentation practices. In particular, tender scar was recorded in 78.7% of Group 5 CSs, a pattern that warrants local review because it may reflect clinical concern, a low threshold for repeat CS, or documentation culture. Oligohydramnios was also recorded among CSs, but isolated oligohydramnios should not be interpreted as a universal indication for CS. Future work should use standardized definitions and blinded peer review of selected records, including CTG and operative documentation, to assess consistency and appropriateness.

### Comparison with other studies

The predominance of Group 5 is consistent with Robson’s analyses from Brazil and France, while Nigerian and Australian reports illustrate that group-specific contributions vary substantially by setting and referral level [17–20]. Guidance on birth after previous CS supports systematic assessment and counseling for eligible women [21], and studies of childbirth preference highlight the importance of patient-centered counseling and education [22]. In the Polish two-center study recommended during peer review, Group 5 was the largest contributor to CSs in both reference-level centers, supporting the use of TGCS as an audit and benchmarking framework [23]. Differences between studies may reflect case-mix, referral level, induction policy, availability of TOLAC, provider thresholds, and local medico-legal context. The relatively large Group 8 and Group 10 in our cohort are compatible with a tertiary referral setting, but they also emphasize the need for future multicenter comparisons within Egypt.

### Strengths and limitations

The strengths of this study include prospective data collection, a large cohort, use of a standardized data sheet, complete classification of all eligible women into Robson groups, and raw-data rechecking during revision. The study provides a baseline for future institutional monitoring using an internationally recognized system.

Several limitations should be acknowledged. First, this was a single-center tertiary-hospital audit over six months, which limits generalizability and may over-represent high-risk groups. Second, historical five-year Robson-classified trends were not available; the study should therefore be viewed as a baseline implementation for future longitudinal monitoring. Third, maternal and neonatal outcomes, body mass index (BMI), socioeconomic status, education, and detailed referral pathways were not captured. Fourth, TOLAC eligibility and counseling were not prospectively recorded, preventing estimation of the proportion of women in Group 5 who were suitable for TOLAC. Fifth, CS indications were recorded as documented by the treating team; multiple factors could be recorded for the same CS, and no independent blinded validation was performed. Missing indication data occurred in 180 CSs (5.3%). These limitations mean that the study describes the structure of CS use but cannot determine whether individual CSs were clinically justified.

### Future research and quality-improvement directions

Future work should include longitudinal Robson monitoring, multicenter Egyptian benchmarking, standardized indication definitions, peer review of selected CS records, and linkage of Robson groups with maternal and neonatal outcomes. Digital tools or a dedicated Robson data-entry module could reduce reporting errors and support high-fidelity implementation. Quality-improvement priorities include auditing primary CS decision-making in Groups 1–4, indications and outcomes of induction in Groups 2a and 4a, pre-labor CS in Groups 2b and 4b, TOLAC eligibility and counseling in Group 5, and the clinical heterogeneity of Group 10.

## Conclusions

This prospective study established the first Robson TGCS baseline at El-Shatby Maternity University Hospital. The high overall CS rate was mainly structured by a large Group 5, high CS rates in Groups 2 and 4, and a substantial contribution from Group 10. The findings support targeted audit of primary CS, pre-labor CS, induction practices, TOLAC eligibility and counseling, and preterm CS decision-making. Because the study was descriptive and did not assess indication appropriateness or maternal and neonatal outcomes, the results should be used to guide further audit and quality improvement rather than to infer causality or judge individual CS decisions.

## Supplementary Information

Supplementary Material 1.

## Data Availability

De-identified participant-level data, the data dictionary, and the analysis protocol that support the findings of this study are available from the corresponding author (A.A.) upon reasonable request. Requests should include a brief research proposal and evidence of institutional ethics approval. A standard Data Use Agreement (DUA) will be required prior to sharing. Access will be granted for research purposes only. Where institutional or legal restrictions prevent the sharing of full datasets, aggregate summary tables will be provided.

## Abbreviations

APH: Antepartum hemorrhage
BMI: Body mass index
CI: Confidence interval
CPD: Cephalopelvic disproportion
CS: Cesarean section
CTG: Cardiotocography
FWA: Federal Wide Assurance
IRB: Institutional Review Board
PET: Preeclampsia
SPSS: Statistical Package for the Social Sciences
TGCS: Ten-Group Classification System
TOLAC: Trial of labor after cesarean
VBAC: Vaginal birth after cesarean
VD: Vaginal delivery
WHO: World Health Organization
