# Supplementary material for: Implementation of Robson’s ten-group classification system for cesarean section rates at a tertiary university hospital in Egypt: a prospective study
Source: BMC Pregnancy Childbirth. 2026 Jul 23;26:804. doi: 10.1186/s12884-026-09693-y (PMC13393242; doi:10.1186/s12884-026-09693-y)
Supplement: Supplementary file 1 — Supplementary Material 1. [file 12884_2026_9693_MOESM1_ESM.pdf]

Implementation of Robson's 10-Group Classification System for Cesarean Section Rates

Part 1: Maternal Characteristics

- **Serial Number:** [ ]

- **Maternal Age (years):** [ ]
- **Parity:**
  - Nulliparous (0)
  - Multiparous (≥1)
- **History of Previous Cesarean Section:**
  - No
  - Yes (Number of previous CS: ___)

Part 2: Obstetric Characteristics (Current Pregnancy)

- **Gestational Age (weeks):** [ ]
- **Number of Fetuses:**
  - Singleton
  - Multiple (Twins/Triplets)
- **Fetal Presentation:**
  - Cephalic
  - Breech
  - Transverse / Oblique
- **Onset of Labor:**
  - Spontaneous
  - Induced
  - Pre-labor Cesarean Section

Part 3: Delivery Outcomes

- **Mode of Delivery:**
  - Vaginal Delivery
  - Cesarean Section
- **Indication for Cesarean Section (if applicable):**

- ---

Part 4: Robson Classification

- **Assigned Group (1–10):** [ ]
